# Drought-induced changes in flow regimes lead to long-term losses in mussel-provided ecosystem services

**DOI:** 10.1002/ece3.1442

**Published:** 2015-02-25

**Authors:** Caryn C Vaughn, Carla L Atkinson, Jason P Julian

**Affiliations:** 1 Oklahoma Biological Survey, Department of Biology, and Ecology and Evolutionary Biology Graduate Program, University of OklahomaNorman, Oklahoma; 2 Department of Biological Sciences, University of AlabamaTuscaloosa, Alabama; 3 Department of Geography, Texas State UniversitySan Marcos, Texas

**Keywords:** Biofiltration, drought, ecosystem service, environmental flows, flow regime, freshwater mussel, nutrient cycling, nutrient storage

## Abstract

Extreme hydro-meteorological events such as droughts are becoming more frequent, intense, and persistent. This is particularly true in the south central USA, where rapidly growing urban areas are running out of water and human-engineered water storage and management are leading to broad-scale changes in flow regimes. The Kiamichi River in southeastern Oklahoma, USA, has high fish and freshwater mussel biodiversity. However, water from this rural river is desired by multiple urban areas and other entities. Freshwater mussels are large, long-lived filter feeders that provide important ecosystem services. We ask how observed changes in mussel biomass and community composition resulting from drought-induced changes in flow regimes might lead to changes in river ecosystem services. We sampled mussel communities in this river over a 20-year period that included two severe droughts. We then used laboratory-derived physiological rates and river-wide estimates of species-specific mussel biomass to estimate three aggregate ecosystem services provided by mussels over this time period: biofiltration, nutrient recycling (nitrogen and phosphorus), and nutrient storage (nitrogen, phosphorus, and carbon). Mussel populations declined over 60%, and declines were directly linked to drought-induced changes in flow regimes. All ecosystem services declined over time and mirrored biomass losses. Mussel declines were exacerbated by human water management, which has increased the magnitude and frequency of hydrologic drought in downstream reaches of the river. Freshwater mussels are globally imperiled and declining around the world. Summed across multiple streams and rivers, mussel losses similar to those we document here could have considerable consequences for downstream water quality although lost biofiltration and nutrient retention. While we cannot control the frequency and severity of climatological droughts, water releases from reservoirs could be used to augment stream flows and prevent compounded anthropogenic stressors.

## Introduction

Fresh water is vital for both humans and fish and wildlife, but humans are using fresh water more rapidly than it can be replenished (Baron et al. 2002). Until recently, issues with sustainable water use in the United States have been associated primarily with the arid southwest (Sabo et al. 2010), but growing human populations and increases in drought frequency and magnitude have raised concerns about future water supplies even in moist temperate areas such as the southeastern United States (Pederson et al. 2012). Because of increasing human demand for freshwater, coupled with impending climate change and subsequent shifts in the duration and frequency of droughts and associated alterations in stream flows, trade-offs between water security for human needs and biodiversity conservation will only become more challenging in the future (Milly et al. 2005).

Ecosystem services are the benefits that humans derive from healthy ecosystems (Perrings et al. 2011; Wainger and Mazzotta 2011). Biologically complex freshwater ecosystems provide important ecosystem services such as provisioning of freshwater, nutrient processing and water filtration, and recreation and ecotourism (Brauman et al. 2007; Dodds et al. 2013). Freshwater mussels (Bivalvia: Unionoida; hereafter “mussels”) provide many important ecosystem services in rivers. Adult mussels typically occur as dense, speciose aggregations called mussel beds (Strayer 2008). Recent work has shown that mussel beds create biogeochemical hot spots (areas with disproportionately high exchanges of reactive materials (McClain et al. 2003)) in rivers (Atkinson and Vaughn 2015). While mussels remove seston through filter-feeding creating top-down effects in streams (Vaughn et al. 2008), they also have strong bottom-up effects in streams via nitrogen excretion (Atkinson et al. 2014b; Strayer 2014) leading to increases in benthic algae (Spooner and Vaughn 2012), macroinvertebrates (Vaughn and Spooner 2006; Spooner et al. 2012), fish (Sansom 2013), and riparian spiders (Allen et al. 2012). Mussel tissue (soft and shell) provides long-term nutrient storage, which in turn alters nutrient limitation and decreases movement of nutrients downstream (shortened nutrient spirals) (Atkinson et al. 2013). Mussel shells also provide biogenic habitat for other organisms (Spooner et al. 2012). Recent work experimentally tracking mussel-derived nitrogen through a stream food web with ^15^N showed that mussel excretion can account for 40–74% of the total N demand in small streams where mussels are abundant (Atkinson et al. 2014b). Effects of mussel-provided nutrients are spatially patchy because of the patchy distribution of beds and temporally variable due to seasonal changes in hydrology and water temperature (Atkinson and Vaughn 2015).

Mussels are one of the most threatened faunas globally, largely because their life-history traits make them highly vulnerable to habitat destruction and alteration, population fragmentation, and introduction of non-native species (Haag 2012). Adult mussels are largely sedentary burrowers; movements are seasonal and on a scale of a few to an estimated maximum of 100 meters (Waller et al. 1999; Kappes and Haase 2012). Thus, unlike mobile stream organisms such as fish and aquatic insects, mussels have limited refugia from disturbance events such as droughts and floods (Sousa et al. 2012; Collas et al. 2014). Mussels are long-lived in comparison with most other stream organisms, with average life spans ranging from 15 to 40 years (Haag 2012). In addition, many species have delayed reproduction and typically do not reproduce until after age 4 (depending on species), leading to long population turnover times (Haag 2012). Consequently, most mussels cannot recover rapidly from disturbance. Finally, mussels are thermoconformers whose physiological processes are constrained by water temperature within species-specific thermal preferences (Spooner and Vaughn 2008; Pandolfo et al. 2012). Thus, changes in water temperature, including those caused by altered flow regimes, can lead to population declines, shifts in community structure, and changes in rates and magnitudes of ecological processes provided by mussel communities (Haag and Warren 2008; Spooner and Vaughn 2008; Galbraith et al. 2010; Spooner et al. 2011; Atkinson et al. 2014a).

Changing climate conditions and population growth are putting increasing pressure on aquatic systems in the south central United States. Extreme hydrometeorological events such as droughts and heat waves are becoming more frequent, more intense, and more persistent (NCADAC 2013). At the same time, water demands from the region's rapidly growing metropolitan areas (e.g., Dallas-Fort Worth, Oklahoma City) have exceeded local supplies (NRDC 2013). In searching for new water sources, both northern Texas and central Oklahoma have focused on the relatively pristine rivers of southeastern Oklahoma. These rivers are known for their high aquatic biodiversity and exceptional water quality (Matthews et al. 2005). However, they are vulnerable to climate warming because they are shallow with high rates of evapotranspiration and are fed predominantly by precipitation runoff (Covich et al. 1997). Further, aquatic organisms such as fish and mussels cannot migrate northward due to prevailing west-to-east drainages, or to higher elevations due to intermittency of headwaters (Matthews and Zimmerman 1990). While periodic heat waves and drought are normal in this region (Stambaugh et al. 2011), human-engineered water storage and management are new phenomena leading to broad-scale changes in flow regimes (Poff et al. 2007).

Here, we ask how observed changes in mussel biomass and community composition resulting from drought-induced changes in flow regimes might lead to changes in river ecosystem services. Our study focused on three mussel-provided ecosystem services: biofiltration, nutrient recycling (nitrogen and phosphorus), and nutrient storage (nitrogen, phosphorus, and carbon), because they have been shown to be ecologically important (Vaughn 2010; Newton et al. 2011), can be quantified (Spooner and Vaughn 2008), and can be compared to similar, human-engineered services (North et al. 2010; Higgins et al. 2011).

## Methods

### Study site and water conflict

**Figure 1:**
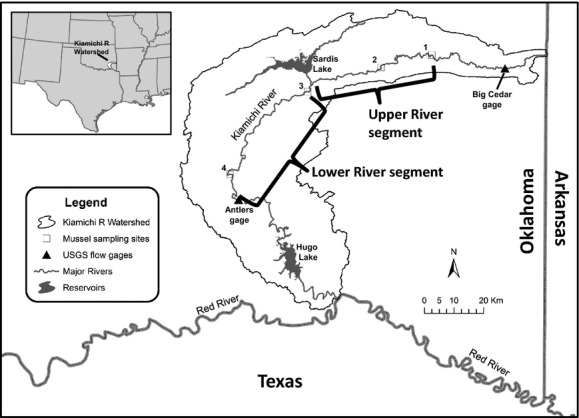
Map of the Kiamichi River showing sample sites, gage locations, and reservoirs. The Upper River segment extends from the town of Whitesboro to immediately above the Sardis Lake confluence, and the Lower River segment is from just below the Sardis Lake confluence to where the river flows into Hugo Lake. The town of Whitesboro is located north adjacent to sampling site 1, and the town of Antlers is located south adjacent to the Antlers USGS gage.

The study was conducted in the Kiamichi River, a fifth-order major tributary of the Red River in southeastern Oklahoma, USA. (Fig.1). The river is known for its high aquatic biodiversity including 86 fish and 31 freshwater mussel species, including three mussels that are federally listed (Master et al. 1998; Matthews et al. 2005; Galbraith et al. 2008). The river arises in the Ouachita Uplands and flows 197 km through a narrow, mainly ridge-and-valley watershed (3686 km^2^). As of 2006, the watershed was 70% forest, 15% agriculture (almost all low-density pasture), 7% grassland/shrubland, 3% urban, 2% water, and 1% other (Fry et al., 2011). The only major change in land cover since 1992 was a 2% increase in grassland/shrubland at the expense of forest (NLCD, 2006). While most of the watershed is temperate deciduous forest, there is conifer logging in the uppermost watershed. The steep watershed has prevented major row-crop agriculture. There are no interstate highways or major cities, and human population density is low (<5 people/km^2^ according to the 2010 U.S. population census) and has not changed appreciably since the 1990 census.

Water from the Kiamichi River is desired by multiple stakeholders for various uses, including Oklahoma City, the State of Oklahoma, the Tarrant County Water District (Fort Worth, TX), local residents in southeastern Oklahoma, and the Choctaw and Chickasaw Nations. Accordingly, there has been conflict and debate over how the Kiamichi waters should be used and governed. At the heart of this controversy is water held by Sardis Dam (completed in 1983), which impounds Jackfork Creek, a major tributary of the Kiamichi River that drains 712 km^2^. This drainage area accounts for 24% of the runoff for the Kiamichi River at the Antlers gage (Fig.1). The Kiamichi River is smaller and higher gradient above the Jackfork Creek confluence, and reaches above and below this confluence are affected differently by water management (Galbraith et al. 2010; Allen et al. 2013). In particular, the downstream reach's flow regime can be dictated by releases from Sardis Dam, especially during summer droughts when dam releases are the only source of flow. In recent drought years, water releases from Sardis Dam during hot summer months have been minimal or nonexistent, contributing to patchy drying of the lower river (Galbraith et al. 2010; Allen et al. 2013; Atkinson et al. 2014a).

We used the Antlers gage (Fig.1) as the downstream extent of our study area here because most mussels are located upstream of this point. While there were historically many large mussel beds below Antlers, most of the river below this point is no longer suitable habitat because of impoundment effects from downstream Lake Hugo (dam completed in 1974).

### Hydrology data

We assessed the flow regime of the Kiamichi River using a network of gages that captured releases from Sardis Dam and discharges above and below this confluence (Fig.1). Daily discharge data (hydrologic year of 1 October–30 September, 1966–2012) for the downstream extent of our study area were collected and analyzed from U.S. Geological Survey (USGS) gage 07336200 (Kiamichi River near Antlers, OK, 1972–2012) and its predecessor gage 07336500 (Kiamichi River near Belzoni, OK, 1966–1972), which was moved upstream to Antlers when Hugo Lake began filling. Discharges at Belzoni were area-corrected to correspond to Antlers measurements. Daily discharge data (1966–2012) from USGS gage 07335700 (Kiamichi River near Big Cedar, OK) were used to characterize the upper segment of the river above the Sardis Dam confluence (Fig.1). Releases from Sardis Dam were assessed using daily data (1995–2012) from the U.S. Army Corps of Engineers gage CYD02.

“Severe hydrologic drought” was defined as flows below the 10th percentile of flow frequency (sensu Svoboda et al. 2002). This threshold also corresponds to “extreme low flows” in flow regime analyses such as indicators of hydrologic alteration (IHA; Richter et al. 1996). We used IHA analyses to characterize and compare flow regimes of downstream versus upstream gages, including the metrics: median flow, days of no flow, and days in extreme low flow. While days of no flow is an absolute measure, days in extreme low flow (also severe hydrologic drought) is a relative measure at each gage based on its entire flow record.

### Mussel sampling

In the early 1990s, long-term mussel monitoring sites were established on the Kiamichi River (Vaughn and Pyron 1995). Sites with major mussel beds were chosen from this data set which were located both upstream and downstream of the confluence with the Sardis Lake outflow. In this study, we used long-term data from four sites, two above and two below the Sardis Lake confluence (Fig.1), that were sampled across three periods: 1992, 2003, and 2011. We chose these sites because we had robust data for all time periods; because one of us (Vaughn) participated in each sampling event, we were confident that sampling was comparable across time periods. At each site for each sampling period, we excavated 15 randomly placed, 0.25-m^2^ quadrats to a depth of approximately 15 cm following Vaughn et al. (1997). Mussels were brought to shore, their length measured, and returned to the mussel bed alive. We used ANOVA (SPSS ver. 19 Armonk, NY, US: IBM Corp.) to compare mussel densities at the four sites over time.

**Figure 2:**
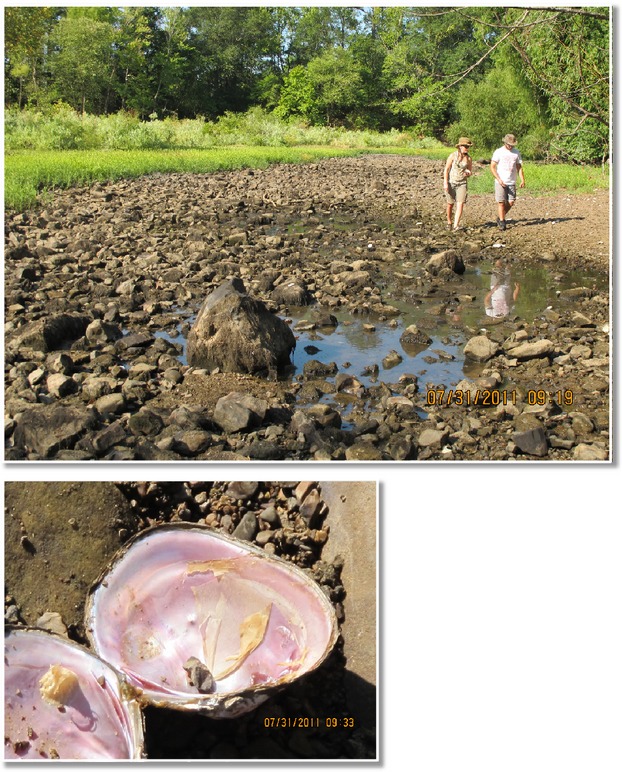
Photographs of site 4 on July 31, 2011, showing dry riverbed and freshly dead mussels.

Additionally, we conducted more intensive sampling at site 4 during extremely low flow conditions in the summer of 2011. When we arrived at this site on July 31, 2011, we discovered that approximately the lower one-third of the mussel bed (87 m in length) was completely dry with many freshly dead (tissue still attached) mussels (Fig.2). We divided the site into three sections: the upstream pool, the downstream riffle that still had some water (hereafter “wet riffle”), and the most downstream riffle that was completely dry (hereafter “dry riffle”). In the pool and wet riffle sections, we excavated 15, 0.25-m^2^ quadrats and identified and measured mussels as described above. In the wet riffle section, there were many freshly dead mussels, so we separately tallied densities and sizes for live and dead mussels. In the dry riffle, we established eight transects across the riverbed spaced 10 meters apart. At each one-meter interval across each transect, we counted freshly dead mussel individuals that could be observed from the surface for one meter to either side of the transect line. We used ANOVA (SPSS ver. 19) to compare mussel densities in the pool versus wet riffle and live versus dead mussels in the wet riffle.

### Estimation of mussel-provided ecosystem services

Our study examined three mussel-provided ecosystem services: biofiltration, nutrient recycling (nitrogen and phosphorus), and nutrient storage (nitrogen, phosphorus, and carbon). To facilitate river-wide comparison of these services across the three sampling periods, we divided the river into two segments: *Upper River* (54.6 km), from the town of Whitesboro to immediately above the Sardis Lake confluence, and *Lower River* (105.4 km), from just below the Sardis Lake confluence to the town of Antlers (Fig.1). We divided the river in this way because the river is smaller, higher gradient, and naturally contains fewer mussel beds above the Sardis Lake confluence than below it. The upper segment begins at the town of Whitesboro (site 1 in Fig.1) because mussels have never been abundant in river reaches above this point. The lower segment ends at Antlers because mussels have been largely extirpated below Antlers because of the Hugo Lake impoundment (Vaughn and Pyron 1995) (Fig.1).

In the Kiamichi River, mussels do not occur continuously throughout the river, rather they are found in multispecies aggregations called mussel beds (Vaughn and Pyron 1995). Because of this, if we had averaged mussel densities across entire river segments we would have overestimated mussel ecosystem services. Instead, we conservatively estimated mussel ecosystem services based on the assessed number of mussel beds and the mean densities of mussels in those beds for each river segment. From longitudinal surveys over the past two decades, we have mapped the locations of virtually all of the mussel beds in the Kiamichi River (Vaughn and Pyron 1995; Galbraith et al. 2008; Atkinson and Vaughn 2015). We determined the bed size of each mussel bed. We first determined the extent of the bed by snorkeling over the area containing aggregated mussels and determining where mussel densities declined significantly. We then measured the length and width of the mussel aggregation. Based on these measures, average mussel bed size in the Upper River is 300 m^2^ and average mussel bed size in the Lower River is 1500 m^2^. In the Upper River, there is a mussel bed every 2.53 km, and in the Lower River, there is a mussel bed every 1.68 km, on average. Therefore, for our estimates we assumed 23, 300 m^2^ mussel beds in the Upper River and 63, 1500 m^2^ mussel beds in the Lower River. We used these values multiplied by areal biofiltration, recycling rates, and storage values discussed below to scale our data up from square meters to river segments.

To assign mussel biomass and subsequent biomass-based ecosystem services to mussel beds, we used a typical 12-species community of the most common mussel species in the river (Table1) based on extensive field studies (Vaughn et al. 1996; Galbraith et al. 2008; Spooner and Vaughn 2009; Atkinson and Vaughn 2015). We excluded rare species from our estimates because they make up a small proportion of community biomass and would have a small influence on the ecosystem services estimated here (Vaughn 1997; Spooner and Vaughn 2009). For each of the four sites across the three time periods, we calculated the mean density of the 12 species and their average shell length. We then used mussel shell length–tissue dry mass regressions to calculate mussel biomass (Vaughn et al. 2007), which allowed us to assign a mean biomass to each species at each site.

**Table 1:** Mussel biomass and excretion rates used to estimate nitrogen and phosphorus recycling rates and storage

	Mean soft tissue dry mass (g/m^2^)														
	Upstream sites			Downstream sites			Ammonium excretion (*μ*g L/h/g)			Phosphate excretion (*μ*g L/h/g)			Clearance rate (L/h/g) ×1000		
Species	1992	2003	2011	1992	2003	2011	15°	25°	35°	15°	25°	35°	15°	25°	35°
*Actinonaias ligamentina*	21.00	6.47	0.00	55.08	23.45	38.19	0.479	1.019	1.636	0.261	0.269	0.410	0.452	3.210	2.363
*Amblema plicata*	21.31	5.21	11.68	31.35	12.80	10.31	0.248	0.556	0.766	0.206	0.229	0.211	0.612	1.211	3.572
*Ellipsaria lineolata*	0.00	0.00	0.00	2.62	11.19	2.61	0.366	0.909	1.362	0.239	0.298	0.359	1.000	3.600	6.600
*Fusconaia flava*	3.05	0.78	0.26	3.65	1.51	0.21	0.230	0.829	0.913	0.204	0.326	0.229	0.709	3.423	7.475
*Lampsilis cardium*	7.70	5.89	1.61	4.42	7.49	1.62	0.578	0.988	2.389	0.299	0.539	0.534	1.810	4.707	2.343
*Lampsilis teres*	0.41	1.83	1.09	0.00	0.00	0.00	0.366	0.909	1.362	0.239	0.298	0.359	1.000	3.600	6.600
*Obliquaria reflexa*	0.25	0.00	0.00	0.76	0.00	0.38	0.216	1.041	1.376	0.208	0.200	0.220	0.694	4.010	9.587
*Potamilus purpuratus*	0.58	0.00	0.00	0.93	13.58	2.11	0.366	0.909	1.362	0.239	0.298	0.359	1.000	3.600	6.600
*Ptychobranchus occidentalis*	0.25	2.38	0.54	0.32	0.00	0.00	0.366	0.909	1.362	0.239	0.298	0.359	1.000	3.600	6.600
*Quadrula pustulosa*	5.49	0.75	0.00	9.13	2.38	0.63	0.167	0.757	1.362	0.186	0.305	0.534	2.224	4.989	15.451
*Quadrula verrucosa*	3.94	1.04	0.41	1.64	0.00	0.74	0.784	0.936	1.572	0.239	0.298	0.359	1.000	3.600	6.600
*Truncilla truncata*	0.21	0.00	0.00	0.43	1.23	0.19	0.340	1.041	1.077	0.294	0.311	0.389	0.911	7.603	7.614

For each species, we used laboratory-derived mass-specific filtration rates and nutrient excretion rates to estimate areal rates of biofiltration, and nitrogen and phosphorus recycling (Spooner and Vaughn 2008). Work by our laboratory has shown that laboratory- and field-measured rates for these processes are comparable (Vaughn et al. 2008; Spooner and Vaughn 2012). Different mussel species perform differently at different temperatures; thus, mussel community biofiltration and nutrient recycling rates differ with mussel community composition and water temperature. To account for such seasonal variation in ecosystem services, rates were measured at 15**°**, 25**°**, and 35**°**C. We used clearance rates (the volume of water from which a mussel has filtered all algal particles), measured as change in chlorophyll *a*, to estimate biofiltration. We used ammonia and phosphorus excretion rates to estimate areal nitrogen and phosphorus recycling. For most species, we used the original data on biofiltration and excretion rates estimated by Spooner and Vaughn (2008) for species in our region; for species where rates were unavailable, we measured rates following Spooner and Vaughn (2008) or used an average across all species (Table1). All rates were corrected for container volume and standardized per gram of mussel dry soft tissue (i.e., shell mass was not included). For each site and period, we calculated the soft tissue dry mass of each species per square meter and then multiplied that value by the mass-specific biofiltration or excretion rates to get an areal rate. We used our measured mussel biomass and stoichiometric data from the literature to estimate the amount of nitrogen, phosphorus, and carbon stored in mussel soft tissue and shell as follows: soft tissue 12% N, 3% P, and 50% C; shell 1% N, 0.01% P, and 15% C (Christian et al. 2008; Atkinson et al. 2010).

## Results

### Flow regimes

The upper segment of the Kiamichi River (at Big Cedar gage) is an intermittent stream with an annual mean of 35 no-flow days (Table2). Zero discharge also corresponds to severe hydrologic drought (<10th percentile) at this site. The lower segment (at Antlers) was a perennial river most years before 1983 when Sardis Dam was constructed on one of its main tributaries (Jackfork Creek), with only a few no-flow periods during exceptional droughts. Severe hydrologic drought occurred 27 days per year, on average. This difference in flow permanency between the upper and lower segments was the result of a few major tributaries (like Jackfork Creek) contributing flow to the Antlers gage even during droughts. The completion of Sardis Dam in 1983 was followed by two relatively wet decades, and the Kiamichi River at Antlers remained a perennial river for the most part, with only 75 no-flow days during this 20-year period.

**Table 2:** Flow characteristics of the lower (at Antlers) and upper (at Big Cedar) segments of the Kiamichi River and one of its main tributaries (Jackfork Creek), which was impounded by Sardis Dam in 1983. Ranges (in hydrologic years) correspond to periods prior to the three mussel surveys: 1983–1990, 1992–2003, and 2004–2011. Predam conditions (1966–1982) are included for reference. Median flow for Jackfork Creek before impoundment was estimated using the proportional area–runoff method on the Antlers predam median flow

	1966–1982	1983–1990	1992–2003	2004–2011
Lower Kiamichi River at Antlers gage				
Median flow (m^3^/s)	8.5	10.0	11.7	4.4
Mean annual no-flow days	9.7	0	5.8	31.1
Mean annual days in severe hydrologic drought	27.1	29.4	30.8	65.4
Upper Kiamichi River at Big Cedar gage				
Median flow (m^3^/s)	0.65	0.74	0.93	0.51
Mean annual no-flow days	35.6	50.9	41.1	56.5
Mean annual days in severe hydrologic drought	35.6	50.9	41.1	56.5
Jackfork Creek below Sardis Dam				
Median flow (m^3^/s)	2.0	0	0	0
Mean annual no-flow days	n/a	n/a	262.0	281.9

Several droughts occurred between 2004 and 2011. During this 7-year period, the lower Kiamichi River had no flow for 249 days, which exceeded the total number of no-flow days for the previous 37 years, by 9 days. On 221 of these 249 no-flow days, there were no releases from Sardis Dam. During this same 7-year period, severe hydrologic drought became more frequent in the lower Kiamichi River (mean annual of 65 days) than in the upper segment (mean annual of 56 days) (Table2). Thus, the lack of releases from Sardis Dam during droughts increased the magnitude and frequency of hydrologic drought in the lower segment of the Kiamichi River. This more intensive hydrologic drought in hot, summer months led to patchy drying of the lower river and high water temperatures. In some cases, water temperatures exceeded 40**°**C because of the extremely shallow water and high air temperatures.

### Mussel responses

**Figure 3:**
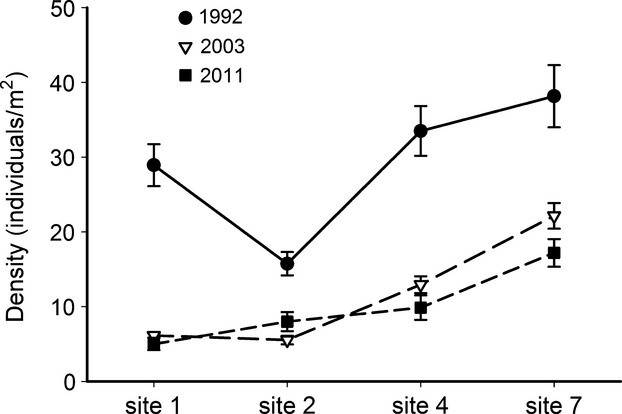
Mean mussel densities (all species combined, ±1 SE) for the four sampling sites over the three time periods. Filled circles 1992, open triangles 2003, filled squares 2011.

Mussel densities declined over time (*F*_2,11_ = 7.43, *P *=* *0.012) (Fig.3). Mussel decline was much steeper between 1992 and 2003 than between 2003 and 2011 (Fig.3). In addition, mussels did not decline site 2 between 2003 and 2011 (Fig.3).

**Figure 4:**
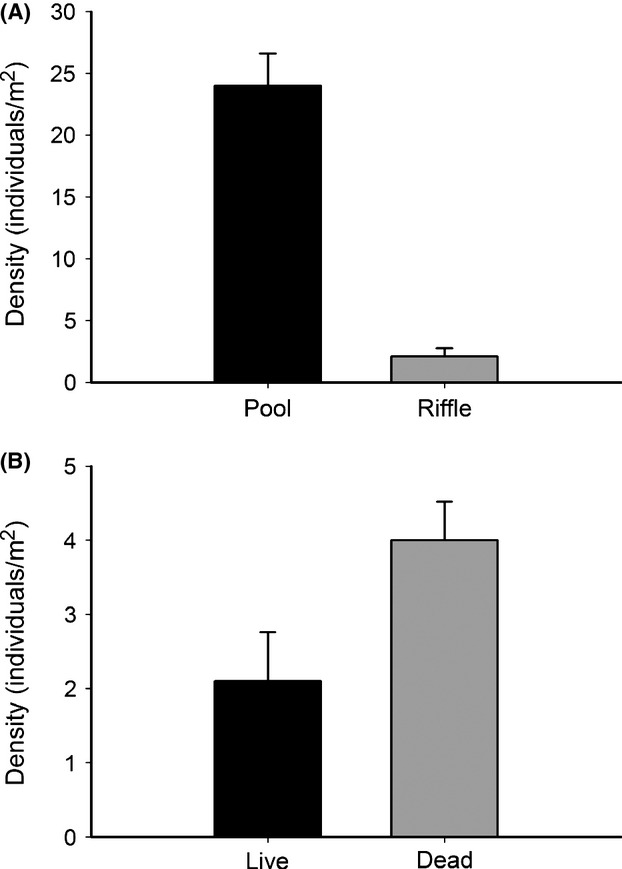
Mean mussel density (±1 SE) at site 4 in 2011. (A) Live mussels in upstream pool versus downstream riffle. (B) Live versus dead mussels in the downstream riffle.

In surveys of site 4 prior to 2011, mussel densities in the pool and riffle portion of the bed have been approximately equal (Vaughn, unpublished); however, in 2011, mussel densities in the pool were approximately 12 times higher than in the shallower wet riffle (Fig.4A, *F*_1,24_ = 37.04, *P *<* *0.001). On July 31, 2011, the pool was covered by water depths of 30-to-100 cm, with midday water temperatures <30°C. In contrast, the portion of the riffle that still had water covering it was extremely shallow (average depth 10 cm) and the midday water temperature was 40°C. In the wet riffle, freshly dead mussels (tissue still attached) were twice as abundant in quadrats as live mussels (Fig.4B, *F*_1,19_ = 6.137, *P *=* *0.023). In the completely dry lower riffle, we found 19 species of freshly dead mussels (Appendix 1).

**Figure 5:**
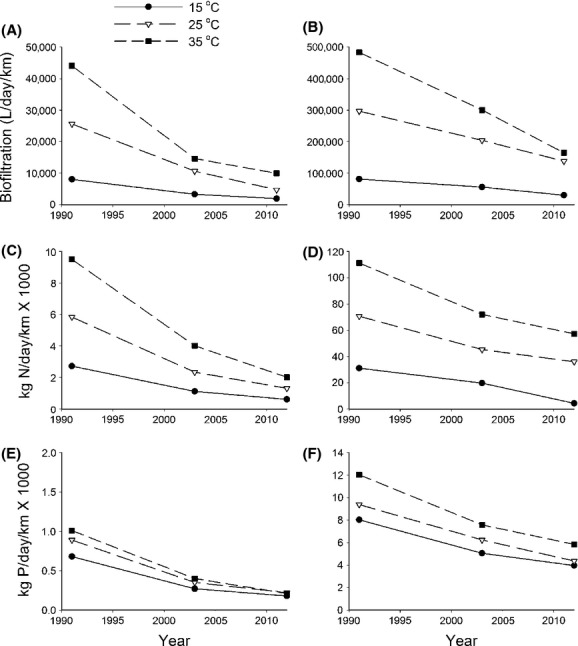
Estimated mussel-provided ecosystem services in the Kiamichi River for three temperature regimes over the three time periods. (A) Upstream biofiltration. (B) Downstream biofiltration. (C) Upstream nitrogen recycling. (D) Downstream nitrogen recycling. (E) Upstream phosphorus recycling. (F) Downstream phosphorus recycling. Filled circles 15°C, open triangles 25°C, and filled squares 35°C.

**Figure 6:**
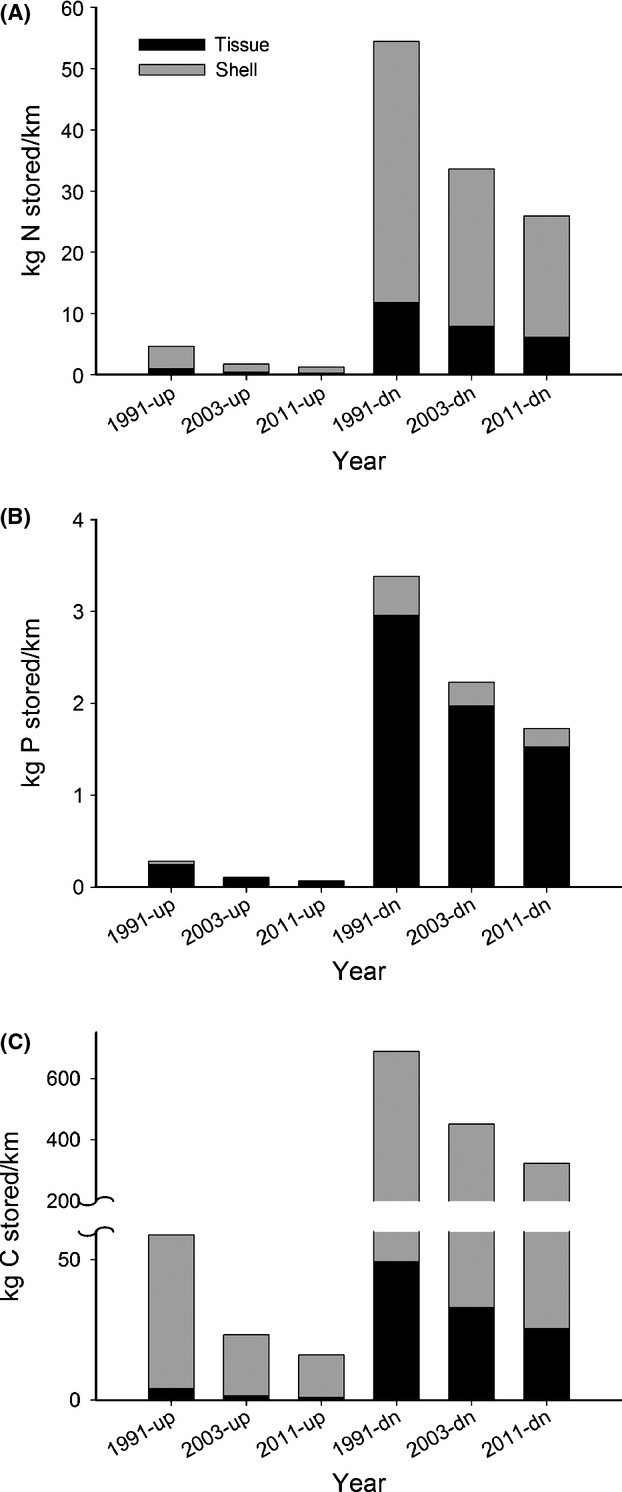
Estimated mussel-provided nutrient storage over three time periods in the Kiamichi River. (A) Nitrogen. (B) Phosphorus. (C) Carbon. Up = upstream, dn = downstream.

Areal biofiltration and nutrient recycling decreased significantly over time and mirrored losses of mussel biomass. The loss of mussel function led to considerable declines in estimated mussel-provided ecosystem services. Declines were steeper in the upstream segment of the river compared to the downstream segment for biofiltration (Fig.5A vs. B), nitrogen recycling (Fig.5C vs. D), and phosphorus recycling (Fig.5E vs. F). However, losses in the downstream segment of the river were of much greater magnitude because mussel-provided ecosystem services in the lower river are an order of magnitude higher than in the upper river. This trend was also displayed by storage losses (Fig.6).

## Discussion

Mussel populations in the Kiamichi River declined over 60% in just 20 years. These biomass losses are catastrophic and equal or exceed highly publicized losses in other ecosystems such as tropical forests (De Beenhouwer et al. 2013), coral reefs (Pandolfi et al. 2003), diadromous fishes (Allan et al. 2005; Limburg and Waldman 2009), and ocean fisheries stocks (Jackson 2008). In our system, mussel biomass losses led to large declines in ecosystem function and major mussel-provided ecosystem services – biofiltration, nutrient recycling, and nutrient storage. Other studies have predicted similar patterns with other taxa. For example, McIntyre et al. (2007) used excretion rates and population sizes of fish species from South America and Africa to predict how species loss would impact system-wide nutrient recycling. However, as far as we are aware, our study is the first freshwater study to link the loss of consumer-provided ecosystem function to ecosystem services that should benefit humans.

In our study, mussel populations never recovered to predrought population levels even though there were several wet periods between 1992 and 2011, likely because this was insufficient time for mussel populations to reproduce and grow, given their relatively long life spans, often delayed time to reproductive maturity and episodic reproduction (Haag 2012). Mussel losses between 2003 and 2011 were less drastic than those before 2003 simply because mussel populations had not recovered from the earlier drought and successful beds were in deeper habitats that did not become dewatered or excessively warm (Fig.4A). Constraints to mussel recovery include time lags associated with their long life span and reduced reproduction and dispersal related to negative density dependence and river fragmentation. Because mussels are long-lived, their densities depend strongly on past as well as current ecological conditions, and it can take decades for them to recover demographically from environmental change (Strayer 2008). Sedentary mussels are spermcasters (Bishop and Pemberton 2006); male mussels release their sperm into the water column and females passively collect the ejected sperm while filter feeding (Galbraith 2009). As mussels decline and populations become smaller, negative density dependence likely leads to fewer sperm finding fecund females and subsequent reduced reproduction (Strayer et al. 2004; Tomaiuolo et al. 2007).

In addition, mussels suffer from an extinction debt (sensu Tilman et al. (1994)) where tributary populations have become isolated from source populations due to habitat destruction and fragmentation (Pringle 2001). Mussels have parasitic larvae that are obligate parasites on fish (Strayer 2008). Because adults are sedentary, the only way that mussels can move between mussel beds within a river or between rivers is as larvae attached to their fish hosts. Many North American large river mussel communities were obliterated during the peak of dam construction in the 1920s–1970s by direct habitat loss (Haag 2009). Mussel populations that have managed to survive in tributaries are isolated from one another by the loss of connecting riverine habitat, such that tributary populations cannot be recolonized by fish hosts (Strayer et al. 2004; Haag 2012). That is, fish cannot transport larvae from populations in other rivers that are no longer connected. Data from the Red River drainage, including the Kiamichi River, support this trend; local extinction rates for mussel populations in the Red River drainage are twice as high as local colonization rates (Vaughn 2012). While extensive, supra-seasonal droughts (Lake 2003) occurred in the Kiamichi River in the past, historically local populations could be rescued from extinction by fish hosts recolonizing from the Red River and its tributaries. This is no longer possible because of the construction of Hugo Lake Dam on the mainstem Kiamichi River just above its confluence with the Red River (Fig.1). Mussel populations in the Kiamichi River above Hugo Lake are isolated, and postdrought recovery can only come from within this reach of river.

Mussel abundance declined dramatically in both the upper and lower river segments. Mussel declines in the upper segment (above Sardis Lake confluence) are most likely due to the effects of multiple droughts because land use has not changed in this segment appreciably over the past few decades. Others studies have documented the high mortality rates and long recovery times exacted by droughts on freshwater mussels, and these losses are usually higher in smaller, shallower streams that are less buffered from changes in temperature and other effects of dewatering (Gagnon et al. 2004; Golladay et al. 2004; Randklev et al. 2013). For example, Haag and Warren (2008) found that mussel abundance declined 65 to 83% in small, southeastern U.S. streams following a severe drought and Shea et al. (2013) documented similar losses in the Flint River basin in Georgia. Although some losses were attributed to direct stream drying (Gough et al. 2012), the majority of losses were due to the secondary effects of low flow, high water temperatures, and high biological oxygen demand (Haag and Warren 2008), much as we have observed in the Kiamichi River (Galbraith et al. 2010). Similar to our results, these studies also found that mussel populations did not have sufficient time to recover between sequential droughts (Fig3; Shea et al. 2013).

We attribute mussel declines in the lower river segment to a combination of long-term drought and human water management. The water now impounded by Sardis Lake historically provided approximately a quarter of the water flowing into the lower river. Following reservoir construction, the lack of releases from Sardis Lake during drought periods has increased the magnitude and frequency of hydrologic drought in downstream reaches (Table2). This increased hydrologic drought in hot, summer months has led to drying of the lower river, high water temperatures (in some cases exceeding 40**°**C because of the extremely shallow water and high air temperatures), and massive mussel mortality (Fig.3). During the same period that we documented the drought-related mussel declines in the Kiamichi River, summer flows were maintained in an adjacent watershed, the Little River, to meet water quality criteria. This river is similar in size and land use, has a similar mussel fauna, and experienced the same hydrometeorological conditions (Matthews et al. 2005; Allen et al. 2013). Mussels in the lower Little River did not decline, which we attribute to managed environmental flows (Allen et al. 2013).

Ecosystem service losses differed with river segment because of the above-described differences in mussel population sizes and water management. The magnitude of ecosystem service declines was greater in the lower river segment, below the Sardis Lake confluence, than in the Upper River segment. This was because biomass losses were greater in the lower segment of the river because it harbors more and larger mussel beds. Ecosystem service losses also varied with mussel tissue type. For example, losses of living mussel tissue led to immediate losses in biofiltration and nutrient recycling capacity (Fig.5). In contrast, losses in nutrient storage capacity (Fig.6) will be slower because of the slow dissolution rate of shells (Strayer and Malcom 2007), which appear to be a significant nutrient sink (Gutierrez et al. 2003; Strayer 2014). Nonetheless, in the short term, ecosystem services in the Kiamichi River have been drastically reduced.

The mussel losses we documented have consequences for stream function. Nutrient excretion by mussels has been shown to alter patterns of nutrient limitation and lead to variation in algal species composition (Atkinson et al. 2013). Furthermore, Atkinson et al. (2014b) enriched mussels with ^15^N and then tracked nitrogen excreted by mussels (mussel-derived N) throughout the food web in the Upper Little River, OK, a watershed adjacent to the Kiamichi River. They found that mussel-derived N met 40 to 74% of nitrogen demand in this segment of river, and mussel-derived N supplied up to 19% of the nitrogen in specific compartments of the food web (primary producers and consumers) near the mussel bed. Thus, the impact of nutrient excretion by mussels is biologically relevant and these losses have considerable effects on stream ecosystem function. In a study of short-term drought effects (2010–2012) in three rivers, Atkinson et al. (2014a) found that mussel declines led to lower nitrogen availability to the food web and reduced phosphorus storage by mussels. In contrast, this study emphasizes the long-term impacts of these losses and shows that there has been little recovery between drought periods.

While the way in which we calculated our biofiltration and nutrient recycling rates has limitations, our estimates are conservative on many levels. We estimated aggregate biofiltration and nutrient recycling rates from measured, species-specific, temperature-dependent physiological rates. Our estimates are likely more realistic for warm temperatures which more closely mimic summer, low flow conditions in the Kiamichi River. We estimated ecosystem services as areal rates and did not take into account seasonal differences in discharge. During summer low flow conditions, mussels in the Kiamichi River can turnover or filter the water column 10 times as it flows over them (Vaughn et al. 2004). However, under higher, winter flows, mussels typically only filter 10% of the water column (Vaughn et al. 2004). To obtain more rigorous estimates of seasonal ecosystem service rates, we need to incorporate discharge into volumetric rate estimates. In addition, we did not measure physiological rates or services at low winter temperatures, as mussel activity at low temperatures is much reduced (Baker and Hornbach 2001; Galbraith and Vaughn 2009). Within these constraints, our estimates of biofiltration are likely conservative because they are scaled up from static, laboratory measures of clearance rates. Marine bivalves and zebra mussels have higher clearance rates in flowing water than under static conditions (Wildish and Kristmanson 1997; Ackerman 1999; Elliott et al. 2008), and this has been recently documented for freshwater mussels (Vanden Byllaardt and Ackerman 2014). Our estimates are also conservative because we only quantified effects of mussels living in large beds and ignored effects of sparser mussel occurrences between beds.

What are the consequences of these lost ecosystem services? Globally, many efforts are underway to restore estuarine bivalve populations because of their documented role in water purification and nutrient fluxes (Newell 2004). For example, oyster reef restoration can significantly increase nutrient removal through increased plankton filtration, increased denitrification rates, and enhanced nutrient sequestration (Cerco and Noel 2010; Higgins et al. 2011; Kellogg et al. 2013; Hoellein and Zarnoch 2014). Carmichael et al. (2012) found that restored oyster populations can remove up to 15% of terrestrial-derived nitrogen loads. In a similar vein, recent work suggests that water extracted for human uses from rivers with healthy freshwater mussel populations may require less treatment than water from rivers without mussels, creating economic benefits (Kreeger and Bushek 2008). Newton et al. (2011) compared the amount of water filtered by mussels in a 480-km reach of the Upper Mississippi River with the amount of water treated by the Minneapolis-St. Paul, Minnesota metropolitan wastewater treatment plant, one of the largest in the Unites States. They found that mussels filtered ∽53 million m^3^/day compared to wastewater flows of 0.7 million m^3^/day, a significantly larger amount. As demonstrated by our results, this substantial biofiltration leads to significant nutrient recycling and storage through mussel growth. Nutrients stored in mussels are retained in the system long term because mussels are long-lived. The remineralized nutrients reduce nutrient spiraling length (Small et al. 2009) and are retained and incorporated into the stream food web rather than being transported downstream (Allen et al. 2012; Atkinson et al. 2013; Atkinson et al., 2014b). While nutrients retained in this manner in one river may seem insignificant, summed across multiple streams and rivers, this biological nutrient retention could help mitigate the effects of nutrient pollution (Vanni et al. 2005; Pilati et al. 2009). Thus, mussel losses like those we document here could have considerable consequences for downstream water quality through lost biofiltration and nutrient retention. Similar effects have been documented for other freshwater consumers such as fishes (Taylor et al. 2006; McIntyre et al. 2007; Bertrand et al. 2009). Further research is needed to quantify ecosystem services provided by mussels, in other watersheds and for additional services such as coupled nitrification–denitrification (Bruesewitz et al. 2008; Hoellein and Zarnoch 2014), and to compare mussel-provided services to human-engineered water treatment.

Drought in the southern plains is cyclical (Stambaugh et al. 2011) and mussels in this region evolved under these conditions. However, drought in this region and the southern United States is predicted to become more frequent and more severe with climate change (Seager and Vecchi 2010), all while the human population is growing and using more water (Sabo et al. 2010). Sustained environmental flows will be especially critical for maintaining ecosystem services during extreme meteorological periods such as droughts, when ecosystems are stressed (Maloney et al. 2012). While reservoirs are now ubiquitous on the landscape, water releases from reservoirs could be used to augment stream flows and prevent compounded anthropogenic stressors (Acreman and Dunbar 2004; Poff and Zimmerman 2010). Cold water releases from dams are already being considered as a management strategy to cool streams and maintain fisheries in the western United States and Australia as they experience climate-induced warming (Cummings et al. 2013; Null and Ligare 2013). Thus, while we have little control over the frequency and severity of climatological droughts, we can control how we manage water resources to maintain populations of freshwater mussels, other stream organisms, and the ecosystem services they provide.

## Appendix 1 Species of freshly dead unionid mussel individuals found at the dry riffle at site 4 on July 31, 2011. We also encountered many freshly dead Asian clams, *Corbicula fluminea*

Actinonaias ligamentina

Amblema plicata

Ellipsaria lineolata

Fusconaia flava

Lampsilis cardium

Lampsilis siliquoidea

Leptodea fragilis

Megalonaias nervosa

Obliquaria reflexa

Obovaria jacksoniana

Potamilus purpuratus

Ptychobranchus occidentalis

Quadrula pustulosa

Quadrula quadrula

Quadrula verrucosa

Strophitus undulatus

Truncilla donaciformis

Truncilla truncata

Villosa iris
